# Effectiveness of mentalization-based therapy and dialectical behavior therapy for cluster B personality disorders: a naturalistic study of service utilization and treatment dropout

**DOI:** 10.3389/fpsyt.2026.1732940

**Published:** 2026-03-02

**Authors:** Phillip Yin, Simon Poirier, Arnaud Allary, Frédéric Pérusse, Pierre David, François-Samuel Lahaie, Lionel Cailhol

**Affiliations:** 1 Department of Psychiatry, University of Montreal, Montreal, QC, Canada; 2 Department of Psychiatry and Addictology, University of Montreal, Montreal, QC, Canada; 3 Institut Universitaire en Santé Mentale de Montréal, Service des troubles relationnels et de la personnalité, Montreal, QC, Canada; 4 Centre Intégré Universitaire de santé et de services sociaux (CIUSSS) Mauricie et Centre-du-Québec, Shawinigan, QC, Canada; 5 Department of Psychiatry, Centre de Recherche de l’Institut Universitaire de Santé Mentale de Montréal, Montreal, QC, Canada; 6 CERVO Research Center, Quebec, QC, Canada

**Keywords:** borderline personality disorder, cluster B personality disorder, dialectical behavior therapy, mentalization-based therapy, psychotherapy, hospitalization, drop-out, emergency department

## Abstract

**Introduction:**

Mentalization-based therapy (MBT) and dialectical behavior therapy (DBT) are effective treatments for cluster B personality disorders (PDs), but few studies have assessed their real-world clinical outcomes in routine practice outside a controlled trial setting.

**Methods:**

Our descriptive naturalistic retrospective study evaluated 288 patients with cluster B PDs who predominantly had borderline PD referred to MBT or DBT.

**Results:**

Observed changes in emergency department (ED) use and hospitalizations one year before and during the first year of therapy were described for patients with at least one relevant event, along with dropout rates. ED visit analyses concerned 104 patients, and hospitalization analyses concerned 30 patients. Across both treatment modalities, ED visits decreased from 119 in the year prior to treatment to 37 during the first year of treatment (p < .001 for both). Hospitalizations were observed to decrease for patients in MBT (p < .05), while no clear change was seen in the DBT group (p = .595). Drop-out rates during treatment were around 30% in both modalities.

**Discussion:**

These patterns descriptively suggest that both therapies are associated with reduced service use during treatment in clinical practice. Future research should investigate which patient- and system-level characteristics can guide patients and clinicians toward the most suitable treatment for everyone, and whether these observed patterns persist beyond the treatment period.

## 1 Introduction

Cluster B personality disorders (PDs) (borderline, narcissistic, antisocial, and histrionic) represent around 13% of the clinical psychiatric population and roughly 2.5% to 3% of the general population (1, 2). Within this group, borderline personality disorder (BPD) is the most prevalent diagnosis in clinical settings. Cluster B PDs are not only common in both general and clinical populations, but they are also associated with a significantly reduced life expectancy, by approximately 9 years for women and 13 years for men at age 20, primarily due to elevated suicide rates and comorbid physical illnesses (1). People with cluster B PDs are also high health care service users: in one year, 78% of them have consulted a family physician, 62% of them visited a psychiatrist, 44% were admitted into the emergency room, and 22% were hospitalized (1). This vital health care use translates into high treatment costs, ranging from $15,000 USD to $50,000 USD per patient every year (3, 4).

Furthermore, many studies have shown that PDs are often comorbid, particularly with borderline personality disorder (BPD) (2, 5). While studying BPD in isolation through randomized controlled trials helps optimize sample homogeneity and design targeted psychotherapeutic interventions, clinicians in real-world settings must frequently manage significant comorbidity, especially among Cluster B PDs. Furthermore, patients with comorbid PDs tend to have a poorer prognosis and are less likely to achieve complete symptomatic remission (6). Therefore, given the high mortality rates, healthcare service utilization, and unfavorable prognosis for patients with comorbid cluster B PDs, it is essential to study this population as a whole.

Mentalization-based therapy (MBT) and dialectical behavior therapy (DBT) are two evidence-based treatments designed for individuals with borderline PD (7, 8). MBT is grounded in psychodynamic theory and attachment theory, with a specific focus on mentalization (9). DBT is a type of cognitive-behavioral therapy that integrates Eastern mindfulness practices to enhance a person’s ability to regulate distress, accept experiences, and manage interpersonal emotions (10). While meta-analyses have established that these treatments have a moderate effect size (Setkowski et al., 2023), their implementation in real-world clinical settings presents several challenges. Patient populations are more heterogeneous, comorbidities are frequent, and therapists differ in their level of experience and training, especially in managing the clinical complexity of Cluster B PDs. Thus, although their efficacy is well established—and even explored in real-world settings for patients with borderline PD (11)—their efficiency in a broader PD population remains underexplored. Similarly, beyond the documented efficacy of specialized psychotherapies in research settings, treatment dropout has been the subject of numerous studies for borderline PDs (12). However, none have specifically focused on the population of individuals with Cluster B PDs.

In this retrospective, naturalistic study, we examine the outcomes of MBT and DBT as delivered in routine clinical practice for adult patients with predominantly BPD along with other Cluster B PDs. Specifically, we aim to describe pre-post differences of these treatments in a real-world setting by analyzing two key clinical indicators during the first year of therapy: the number of emergency department (ED) visits and psychiatric hospitalizations. These ‘hard outcomes’ are frequently used in the literature to evaluate clinical trajectories, with reductions typically reflecting a more favorable prognosis (11, 13, 14). Additionally, we investigate treatment completion rates by comparing dropout rates before and after the start of therapy. Given the naturalistic design, absence of a control group, and lack of *a priori* hypotheses, all findings are interpreted as associative and exploratory rather than as evidence of treatment effects or comparative effectiveness.

## 2 Methods

### 2.1 Study design and participants

This retrospective, naturalistic, and descriptive study was conducted in a real-world, clinical outpatient setting. We examined adults who have been referred to MBT or DBT from general psychiatrists or the ED after being admitted to the “Service des troubles relationnels et de la personnalité”, which is an outpatient clinic at the Institut Universitaire en Santé Mentale de Montréal, in Quebec, Canada, between January 1^st^, 2015, and December 31^st^, 2019. Most participants were diagnosed with at least one cluster B PD as their primary diagnosis. In some cases, patients with a PD not otherwise specified (NOS) or PD traits as their primary diagnosis were also admitted if it caused them significant distress or daily life dysfunction. The study’s population consisted of 363 participants, among whom 75 had dropped out before entering treatment modalities and were excluded from further analysis. The final treatment cohort consisted of 288 patients, which was used for descriptive analyses of treatment dropout following initiation. Analyses of emergency department visits and hospitalizations were further restricted to subgroups of patients who presented with at least each respective event in the observation period. All patient data were extracted from the DATA Bank, which includes all voluntary patients admitted to the outpatient clinic. Both the data bank and this specific study were reviewed and approved by the Research Ethics Board of the CIUSSS de l’Est-de-l’Île-de-Montréal., with which the Institut Universitaire en Santé Mentale de Montréal is affiliated, and the procedures followed were by the Helsinki Declaration as revised in 2013. Written informed consent for the use of clinical data was obtained from patients at admission, and the ethics board waived the requirement for additional consent specific to this retrospective analysis.

Participants were included under the following conditions:

-They were diagnosed with at least one cluster B PD, or a PD NOS with cluster B personality traits, or PD traits with significant distress and daily life dysfunction
-They were oriented to MBT or DBT upon admission to the outpatient clinic.

There were no exclusion criteria for this study.

### 2.2 Procedure

The participants were screened and included in the study based on criteria from the DSM-IV, DSM-5, the Structured Clinical Interview for DSM-IV Axis II (SCID-II), and the Borderline Personality Questionnaire (BPQ). They were not randomized to treatment. Orientation to MBT or DBT was based on the assessor’s clinical judgment and experience, considering the patient’s preferences and available program capacity. As such, differing baseline clinical or demographic characteristics between the two groups may reflect this allocation.

For clinical purposes and data collection, the first group therapy meeting was considered to be the start date of the treatment for MBT and DBT. Consequently, participants who dropped out before the first group therapy session, such as during pretreatment or on the waiting list, were excluded from the analyses examining outcomes during treatment. These individuals were, however, included in descriptive analyses of pre-treatment dropout.

### 2.3 Treatments

Before entering treatment, patients first completed 3–4 psychoeducational group sessions on PDs, followed by 4–6 weekly individual “pretreatment” sessions to prepare them for MBT or DBT. After that, they began one of the two therapies, which is a combination of weekly group and biweekly individual therapy sessions. In both psychotherapies, patients had a treatment contract with precise and measurable objectives. Psychiatric follow-up appointments were also offered as needed. A patient could only continue the treatment on the condition that they were motivated to attend and be engaged in an active social role, such as school, work, or volunteering.

MBT lasts up to two years. The treatment contract is renewed at the end of every module (each 6 months) based on a joint discussion about efficacy between the individual therapist, one group therapist, and the patient. In contrast to Bateman and Fonagy’s original MBT, our sessions were held on a biweekly basis instead of a weekly basis, to allow more patients to be in active treatment. Another difference is the inclusion of four introductory sessions of explicit mentalization at the beginning of each group therapy module, followed by implicit mentalization for the rest of the module. This has been a modification from the original complete therapy module of explicit mentalization before going on to the implicit mentalization therapy modules. Optional post-treatment sessions, consisting of four individual sessions over a six-month period, were offered on a voluntary basis to patients after completion of the MBT. The treatment was provided by two psychiatrists, three psychologists, and two social workers. Among them, six professionals had undergone psychotherapy training, and six members received specific training in MBT. Members of our MBT team had on average 15 years of experience in mental health care (ranging from 1 to 28 years) and on average 7 years of clinical experience specific to PDs (ranging from 1 to 20 years).

DBT treatment in our outpatient clinic lasts one year, but eligible patients can go on for a second year in what is called Graduated Groups. There are some differences between Linehan’s DBT and the DBT clinical services in this study: at our clinic, individual sessions are biweekly (for the same reason as cited above), an emergency phone service is not offered (as we rely on well-organized existing local crisis centers), the mindful meditation module has been reorganized into one on life habits, and each module includes mindfulness exercises. Optional post-treatment sessions (4 individual sessions in 6 months) were offered on a voluntary basis to patients after completion of DBT. At our DBT clinical service, four nurses and three occupational therapists provide the treatment. Members of our DBT team had on average 7 years of experience in mental health care (ranging from 1 to 20 years) and on average 4 years of clinical experience specific to PDs (ranging from 1 to 9 years). One staff member among them had received psychotherapy training, and five had received particular training in Linehan’s DBT.

The therapists for both MBT and DBT were under monthly supervision and attended weekly team meetings to discuss the patients’ treatment status.

### 2.4 Measurement variables

The study descriptively examines observed changes in ED visits and hospitalizations among patients receiving MBT or DBT, as well as their dropout rate following treatment initiation, with treatment initiation defined as the date of the first group therapy session. Only ED visits occurring within the same psychiatric hospital were included.

#### 2.4.1 Emergency department visits

We measured this variable by calculating the observed difference between a participant’s number of ED visits at our psychiatric hospital in the year prior to treatment initiation and during the first year of treatment.

#### 2.4.2 Hospitalizations

We measured this variable by calculating the observed difference between a patient’s number of hospitalizations at our psychiatric hospital in the year prior to treatment initiation and during the first year of treatment. A patient is considered hospitalized if he or she is kept in the hospital for a minimum of 1 day. Usually for these patients, hospitalization was indicated due to a state of crisis or high risk of dangerousness which does not allow them to be referred to an external crisis service.

#### 2.4.3 Dropout rate

It is defined as the percentage of participants who either quit treatment unilaterally without prior discussion with their therapist or whose therapist decided to stop therapy because the treatment conditions were no longer met (for example a lack of attendance). The dropout rate is measured up until one year after the treatment’s start date. Patients who paused or stopped treatment in agreement with their therapist were not considered dropouts (the latter were considered as therapy discontinuation).

### 2.5 Statistical analysis

Analyses were conducted for descriptive and exploratory purposes only, consistent with the naturalistic and hypothesis-generating design of the study. No *a priori* hypotheses or power calculations were specified.

To describe baseline clinical and demographic characteristic differences, independent samples t-tests were used to summarize differences between both patient groups’ age, average mental disorder and PD diagnoses, as well as CGI-S and BPQ scores. Chi-square tests of homogeneity were used descriptively to compare the sex and cluster B PDs distributions.

To characterize observed pre–post differences within each treatment group, paired t-tests were used to summarize changes in ED visits and hospitalizations before and during treatment. Where inferential statistics are reported, they are presented as descriptive indicators of change rather than as formal analyses of treatment effects. For all statistical tests, necessary assumptions were verified to ensure interpretability of descriptive summaries. For calculations regarding ED visits, only participants who had at least one ED visit either before or during the first year of treatment were included. Patients who had never visited the ED either observational period were excluded, as the aim was to describe variation in service use among patients who utilized emergency services at least once. Additionally, since non-users constituted a significant proportion of the patients, analyzing the entire sample would have led to a non-normal distribution, which would have limited the descriptive interpretability of pre-post change estimates. The same logic was applied for hospitalizations, which were therefore restricted to participants who experienced at least one psychiatric hospitalization in either observation period.

## 3 Results

A total of 288 participants began treatment and were included in the descriptive outcome analyses: 130 patients in MBT and 158 in DBT. **Table 1** summarizes the baseline sociodemographic and clinical characteristics of the two groups, as well as the 75 patients who dropped out before treatment began. Notably, most of the participants in both treatment groups were female, with a female-male sex ratio of 1.9 in the MBT group and 10.3 in the DBT group. In the MBT group, 29.2% of participants have a narcissistic PD, as opposed to 4.4% in DBT. Most participants in both groups were single (69.3% in MBT and 71.2% in DBT) and of White origin (90.3% in MBT and 79.5% in DBT). Notable descriptive group differences included age (*p* = .01), sex ratio (*p* <.001), and the prevalence of borderline (*p = .*007) and narcissistic (*p* <.001) PDs. These differences reflect the non-randomized, clinically assigned nature of the treatment allocation.

**Table 1:** Sociodemographic and mental health characteristics of the participants.

Variables				
	MBT group (*N=130*)	DBT group (*N=158*)	P-value (MBT vs DBT)	Pre-treatment characteristics dropout group (*N=*75)
Age at Psychiatric Evaluation (years) Mean (SD)	32.8 (8.6)	30.0 (9.1)	.01	30.2 (8.8)
Sex				
Female N (%)	67 (65.7%)	113 (91.1%)		46 (65.7%)
Male N (%)	35 (34.3%)	11 (8.9%)		24 (34.3%)
Female/Male Ratio	1.9	10.3	<.001	1.9
Ethnicity				
White N (%)	93 (90.3%)	101 (79.5%)		66 (91.7%)
Middle Eastern N (%)	0	8 (6.3%)		0
Native American N (%)	1 (1.0%)	0		0
Black N (%)	1 (1.0%)	3 (2.4%)		2 (2.8%)
Asian N (%)	2 (1.9%)	5 (3.9%)		0
Latin American N (%)	1 (1.0%)	1 (0.8%)		0
Other N (%)	5 (4.9%)	9 (7.1%)		4 (5.6%)
Salary ($ CAD)				
Median bracket	20,000 to 29,999	10,000 to 19,999		10,000 to 19,999
≤ 9,999 N (%)	13 (13.0%)	40 (32.2%)		24 (35.8%)
10,000 to 19,999 N (%)	22 (22.0%)	29 (23.4%)		23 (34.3%)
20,000 to 29,999 N (%)	19 (19.0%)	17 (13.7%)		8 (11.9%)
30,000 to 39,999 N (%)	17 (17.0%)	16 (12.9%)		8 (11.9%)
40,000 to 49,999 N (%)	13 (13.0%)	14 (11.3%)		4 (6.0%)
≥ 50,000 N (%)	16 (16.0%)	8 (6.5%)		0
Marital status				
Married N (%)	3 (3.0%)	2 (1.6%)		2 (2.9%)
Divorced N (%)	1 (1.0%)	5 (4.0%)		1 (1.4%)
Separated N (%)	11 (10.9%)	7 (5.6%)		2 (2.9%)
Single N (%)	70 (69.3%)	89 (71.2%)		50 (71.4%)
Common-law partnership N (%)	16 (15.8%)	20 (16.0%)		15 (21.4%)
Parenthood				
Have children N (%)	36 (36.4%)	48 (42.9%)		24 (36.4%)
No children N (%)	63 (63.6%)	64 (57.1%)		42 (63.6%)
CGI-S score N (%)	4.1 (0.5)	4.2 (0.6)	.160	4.3 (0.6)
BPQ score Mean (SD)	51.0 (10.0)	49.3 (15.6)	.324	52.3 (11.1)
Mental disorder diagnosis				
Zero N (%)	3 (2.3%)	1 (0.6%)		1 (1.3%)
One N (%)	23 (17.7%)	43 (27.2%)		9 (12%)
Two N (%)	37 (28.5%)	40 (25.3%)		14 (18.7%)
Three N (%)	37 (28.5%)	30 (19.0%)		17 (22.7%)
Four or more N (%)	24 (18.5%)	40 (25.3%)		32 (43.8%)
Average N (%)	2.56 (1.33)	2.62 (1.51)	.730	3.41 (1.84)
Personality disorders				
Zero	8 (6.2%)	5 (3.2%)		3 (4%)
One	74 (56.9%)	127 (80.4%)		46 (61.3%)
Two	35 (26.9%)	17 (10.8%)		19 (25.3%)
Three or more	7 (5.4%)	5 (3.1%)		5 (6.7%)
Average	1,33 (0.68)	1.15 (0.53)	.016	1.36 (0.67)
Cluster B	107 (82.3%)	144 (91.1%)	.043	67 (89.3%)
Borderline	101 (77.7%)	142 (89.9%)	.007	66 (88%)
Narcissistic	38 (29.2%)	7 (4.4%)	<.001	16 (21.3%)
Histrionic	0	4 (2.5%)		0
Antisocial	1 (0.8%)	6 (3.8%)		6 (8%)
Cluster A	1 (0.8%)	0		0
Cluster C	14 (10.8%)	13 (8.2%)		7 (9.3%)

M, Mean; SD, standard deviation; CGI-S, Clinical Global Impression - Severity.

**Table 2** summarizes the results concerning pre-post differences in ED visits and hospitalizations, as well as dropout trends for the MBT and DBT groups.

**Table 2:** Outcome measures of the participants after 12 months.

Variables	M (SD) or %	
Emergency department visits	MBT (N=43*)	DBT (N=61*)
Total visits (before treatment)	48	71
Total visits (during treatment)	12	25
Mean Reduction	0.837 (1.090)	0.754 (1.178)
Hospitalizations	MBT (N=15*)	DBT (N=15*)
Total admissions (before)	13	11
Total admissions (during)	4	8
Mean Reduction	0.6 (1.056)	0.2 (1.424)
Dropout rates	MBT (*N=130*)	DBT (*N=158*)
Within 12 months	29.2% (38/130)	31.0% (49/158)
0–3 months	7.7%	16.5%
0–6 months	15.4%	21.5%
0–9 months	21.5%	23.4%
0–12 months	29.2%	31.0%
≥ 12 months	36.9%	--

M , Mean; SD, standard deviation. *Only patients with ≥1 ED visit or hospitalization before or during the first year of therapy were included.

### 3.1 Emergency department visits

Of the 288 participants, 104 had at least one ED visit before or during the first year of treatment and were included in this outcome analysis (43 patients in MBT and 61 in DBT).

For the ED visits of both groups analyzed together, there was an average decrease of 0.788 visits to the ED (*SD* = 1.138), *t*(103) = 7.067, *p* <.001, *d* = 0.693. In absolute numbers, these visits decreased from 119 in the year prior to treatment initiation (48 before MBT and 71 before DBT) to 37 during the first year of treatment (12 after MBT and 25 after DBT).

In the MBT group, an average decrease in ED visits of 0.837 visits per person (*SD* = 1.090) was observed, *t*(42) = 5.039, *p* <.001, *d* = 0.768. Similarly, an average decrease of 0.754 visits per person (*SD* = 1.178) was observed in the DBT group, *t*(60) = 4.998, *p* <.001, *d* = 0.640. These statistics are presented as descriptive indicators of observed change over time rather than as tests of treatment effects.

### 3.2 Hospitalizations

A total of 30 patients had at least a psychiatric hospitalization during the year prior to or during the first year of treatment and were included for analysis (15 in each group).

Regarding hospitalizations for both treatments together, an average decrease of 0.4 hospitalizations per participant (*SD* = 1.248) was observed, *t*(29) = 1.755, *p* = .09. In absolute numbers, hospitalizations decreased from 24 before treatment (13 in MBT and 11 in DBT) to 12 during the first year of treatment (4 in MBT and 8 in DBT).

Within the MBT group, an average reduction of 0.6 hospitalizations per person (*SD* = 1.056) was observed, *t*(14) = 2.201, *p* <.05, *d* = 0.568. The DBT group, on the other hand, had an average decrease of 0.2 hospitalizations per person (*SD* = 1.424), *t*(14) = .544, *p* = .595. Given the small subgroup sample sizes, these observations should be interpreted cautiously and are reported for descriptive purposes only.

### 3.3 Dropout rates

Among patients who began treatment, 87 of the 288 patients (30.2%) dropped out of treatment during the first year. In the MBT group, 38 of 130 patients (29.2%) dropped out, while in the DBT group, 49 of 158 (31.0%) dropped out. These rates are purely descriptive due to significant sociodemographic and clinical differences between the two groups following non-randomized allocation.

When examined in three-month intervals, dropout patterns differed descriptively between modalities. Dropouts occurred at a relatively constant for MBT, with around 8% (or 10 participants) abandoning every 3 months in the first year. The DBT group exhibited a higher concentration of dropout in the first quarter (26 people or 16.5%), with decreasing rates in subsequent intervals.

### 3.4 Pre-treatment dropout

Among all eligible patients, 75 participants (or 20.6%) dropped out before the treatment began, typically during pretreatment sessions or while on the waiting list. These individuals were excluded from the outcome analyses. Descriptively, this group showed similar age and illness severity scores (CGI-S and BPQ) compared to patients who started treatment, but they presented with a higher number of diagnosed mental disorders, with 43.8% having four or more diagnoses, compared to 18.5% in the MBT group and 25.3% in the DBT group. They also had slightly more PD diagnoses on average. From a socioeconomic perspective, none of these patients reported an annual income exceeding $50,000 CAD, and approximately 70% earned less than $20,000.

## 4 Discussion

To our knowledge, this study is among the first to descriptively report real-world outcome patterns on patients with predominantly BPD and other cluster B PDs referred to either MBT or DBT in an outpatient setting. Several descriptive observations emerge from the data. Differences in diagnostic profiles at treatment entry were observed: clinicians tend to refer more patients with narcissistic PD to MBT and those with borderline PD to DBT. These patterns likely reflect clinician judgment within a non-randomized allocation process rather than differential treatment indications. Overall, we observed a decrease in ED utilization in both groups during the first year of treatment. Observed reductions in psychiatric hospitalizations were limited to the MBT group, while no clear change was observed in the DBT group. Finally, while the overall dropout rate during treatment was similar between the two groups, the dropout trajectories differed: it was steady over time in the MBT group but concentrated in the early stages of treatment in the DBT group. Additionally, a substantial proportion of patients dropped out before initiating psychotherapy, particularly those from low-income backgrounds.

First, the observed longitudinal decrease in healthcare utilization aligns with findings from controlled studies in patients with BPD (13, 14). These patterns give rise to the hypothesis that participation in structured, specialized psychotherapy may be associated with reduced reliance on ED services during treatment, even in heterogeneous, real-world populations, by improving crisis anticipation, emotional regulation, and help-seeking behaviors. These therapies may also foster a stronger therapeutic alliance and a sense of containment, allowing acute distress to be managed within planned clinical contacts in therapy sessions rather than through unscheduled emergency visits. The absence of a clear reduction in hospitalizations may reflect multiple factors, including the relatively small number of hospitalization events, the inclusion of a less severely affected population, at least about hospitalization risk, compared to those enrolled in controlled trials, and/or a lack of statistical power. These findings should therefore be interpreted cautiously and as descriptive only. Furthermore, differences in service were only observed before and during the first of year therapy. Further research is needed into determining whether these differences persist after treatment concludes.

Secondly, dropout rates observed after one year were substantial and consistent with those reported in a recent meta-analysis by Arntz et al. (12). Regarding descriptive trends across three-month intervals, our findings similarly showed a steady attrition rate of approximately 7–8% per quarter for MBT, in line with Arntz et al.’s observations (12). Specifically, in the DBT group, the overall dropout rate (31.0%) was higher than in Linehan’s original trials—19% (1991) and 17% (2006)—but comparable to the 36% reported in a UK trial by Zimmerman et al. (15–17).

Notably, dropout in the DBT group was highest in the first quarter (16.5%), reflecting a pattern of early disengagement also reported by Arntz et al., who observed a similar rate of approximately 20% (12). Although the temporal dropout patterns qualitatively differed between treatments, with DBT showing early attrition and MBT a more evenly distributed dropout rate, it is hypothetically plausible that these trends likely reflect structural differences between the therapies, variations in diagnostic and sociodemographic characteristics of the patient groups, and the absence of specific program components in our setting, such as telephone coaching in DBT, which may have contributed to the higher early dropout rate. Future studies specifically designed to examine treatment processes would be required to test these hypotheses.

It is essential to highlight that approximately 20% of participants eligible for either therapy dropped out before beginning their first group session. This group appeared to have greater clinical complexity, with a higher proportion of patients having four or more concurrent mental disorder diagnoses and slightly more PD diagnoses than treatment starters. This pattern may indicate that more clinically complex patients may face greater barriers to engaging in intensive psychotherapy. Additionally, socioeconomic characteristics further distinguished this group. None of these individuals reported an annual income above $50,000 CAD, and approximately 70% earned $20,000 CAD or less. These findings give rise to the hypothesis that socioeconomic factors such as poverty and unemployment may pose significant barriers to engaging in psychotherapy. This is consistent with a systematic review by McMurran et al., which found that treatment non-completion in PDs is associated with unemployment and lower levels of education and occupational status (18). In addition, it is plausible that clinician-level and systemic factors, including implicit biases and service-level selection processes, may contribute to unequal access to care for individuals with low socioeconomic status (SES), further exacerbating mental health disparities. These early dropouts likely introduce a selection bias into real-world effectiveness studies, since the most clinically complex and socioeconomically disadvantaged individuals are systematically underrepresented in analyses. Although the observed differences in our study were relatively modest, increasing awareness of the link between SES and therapy engagement may encourage clinicians to reflect critically on their own referral and selection practices (19).

A key strength of this study is its naturalistic design, which enhances ecological validity by capturing the realities of routine clinical practice.

Nonetheless, several limitations should be noted. The retrospective, non-randomized design limits causal inference and introduces potential confounders such as therapist bias and variability in clinical background. MBT was delivered by clinicians with extensive psychotherapy experience, while a team with less general experience but formal DBT certification implemented DBT. The DBT protocol used was also adapted rather than standardized, reducing comparability with previous trials. Treatment delivery was further affected by disruptions related to the COVID-19 pandemic, particularly for patients who began therapy in late 2019.

Additionally, the reliance on hospital records may have led to underreporting of ED visits and hospitalizations occurring outside our institution. However, the sectorized structure of mental health care in our region likely limited this impact. The sample was primarily composed of individuals with borderline and narcissistic PDs, restricting generalizability to other Cluster B subtypes. The exclusion of non-service users from the ED visits and hospitalization differences may also bias estimates and limits generalizability to a heterogeneous general adult psychiatric population referred for either type of therapy. Finally, although dropout was clearly defined, the absence of systematic documentation of reasons for discontinuation limits our ability to interpret adherence and treatment engagement.

## 5 Conclusion

This naturalistic study highlights that both MBT and DBT, when integrated into routine care for adults with Cluster B personality disorders, are descriptively associated with reduced emergency service use during the first year of treatment despite high dropout rates and differences in patient profiles. These observations underscore the real-world complexities of structured therapies delivered by diverse clinical teams. Future research should prioritize identifying patient-, therapist- and systemic-level predictors of engagement and to advance personalized care for patients with Cluster B personality disorders. Understanding the factors influencing treatment allocation is essential, as is exploring potential sources of stigma, such as socioeconomic disadvantage, that may hinder access to care.

## Data Availability

The raw data supporting the conclusions of this article will be made available by the authors, without undue reservation.
